# Contemporary Theory and Research

**Published:** 1893-09-23

**Authors:** 


### Contemporary Theory and Research.

### SCOPOLAMINE.

M. Bellarminon, privatdocent of ophthalmology at the military academy of medicine at St. Petersburg, has proved by numerous clinical experiments the excellent effects, mydriatic, analgesic, and antiphlogistic of chlorhydrate of scopolamine, recently introduced into the treatment of ocular affections by M. Bahlmann. As the paralysis of the accommodation produced by scopolamine lasts a much shorter time than that produced by atropine, M. Bellarminon considers scopolamine is specially indicated, and should be preferred to atropine in cases where it is desired to determine the state of refraction and the accommodation of the eyes, as well as in certain ocular affections of short duration, such as phlyctene of the cornea, hyperaemia of the iris, or irritations of the eye set up by the introduction of foreign bodies.

### A DANGEROUS REMEDY.

Among new drugs, or rather new preparations, we note cicutine or conicine, extracted from the conium maculatum or hemlock used by the Greeks as the deadliest poison then known for inflicting capital punishment. It will be remembered that Plato gives a description of the deadly paralysing affects of the poison on Socrates, spreading from the feet upwards to the heart, inflicting apparently no pain, and leaving the mind unimpaired till the end. Recently bromhydrate or chlorhydrate of cicutine has been found to possess an anæsthetic, analgesic, and slightly hypnotic action. It affects specially the respiration, which it tends to render slower, and it has a calming effect in neuralgia, asthma, and epilepsy, and other incurable maladies of the nervous system. Yet it has been proved over and over again to be a faithless and dangerous drug, and cannot be employed except in the smallest doses and under the very greatest precautions.

### THE MYOPHONE.

Some account is given in *Nature* of the experiments recently made by M. D'Arsonval on the electric excitability of muscles after death. General opinion on this point agreed that the excitability disappeared very soon after the death of the animal, which is true only so long as one depends on the shortening of the muscle for an indication of its sensibility. But this method is not sensitive enough to indicate disturbances of very small amplitude. For this purpose M. D'Arsonval has for many years used a special modification of the microphone, which he has named the myophone, and which, when it is connected with the muscle under experiment, gives a sound some time before any contraction is apparent, especially if the muscle is stretched by a string. By this means it may be proved that nervous excitability may last for many hours after death. As an instance, the achilles tendon of a rabbit may be attached to the myophone, and the sciatic nerve excited by a current broken some 50 to 100 times per second. Besides proving that the death of a nerve is much less rapid than was hitherto supposed, these experiments also show that nerve may act on muscle without producing actual contraction, but only some simple molecular vibration.

### SIMULTANEOUS MOVEMENTS.

The *Nuova Antologia* relates some researches lately made by Dr. Patrizi on the rapidity with which fatigue is shown when movements, either alternate or simultaneous, are per formed by corresponding members of the body. Observing a gymnast raising rythmically and contemporaneously two dumb-bells, he perceived that after a certain time the agreement between the movement of the two arms tended to be destroyed, and that the left arm fell behind the right. In order to study the phenomenon, he had recourse to two instruments, in which the middle fingers of each hand raised equal weights at a given interval, leaving traces of the course of the weights above on a revolving cylinder. The two fingers worked sometimes together and sometimes alternately, but always with the same interval of time. In all the experiments the force exerted appeared greater when the movements were alternate; in the contemporaneous work, the diminution was due almost entirely to the left hand. In conclusion, Dr. Patrizi observes that supplying simultaneous forces from opposite halves of the body implies the co-existence, and hence the strife of two distinct psychic acts, and attention is diverted from one of these acts to the benefit of the other. But are not these mechanical movements after all a mere question of practice?

### METALLOSCOPY.

This form of treatment, popularly known as "Burqism," from M. Burq who first brought it into notice, has been lately brought forward afresh, and presents many curious phenomena. It has been chiefly, though not exclusively, employed in the case of hysterical patients, and rests on the supposition that every person is intensely susceptible to the action of one particular metal—gold, silver, zinc, iron, &c. Experiments made by M. Charcot led him to discover that the amblyopia or withdrawal of the visual sense for colours, so often observed in hysterical patients, could be overcome by the application of the particular metal to which the patient was most sensitive, and he noticed that the colour sense disappeared according to a constant order—violet went first, then green, then red, and finally blue. Applying a plate of the appropriate metal to the forehead of patients thus affected with amblyopia, he observed that the colour sense gradually returned in inverse order to that of its disappearance, viz., the patient became conscious first of blue, orange, red, then of green, and at length of violet. These and other facts connected with the influence of special metal on peculiar temperaments led to an extension of the system, by the administration of metallic doses. "'Metallothérapie' consists simply in administering to patients these metals internally, for the most part under a soluble form. Thus, for example, for gold a solution of chlorure of gold and of sodium is employed, twenty-five drops enclosing a centigramme of the medicament. It is a transparent solution of a beautiful yellow colour, which patients take very well. If the patient is sensitive to zinc, sulphate of zinc is administered in the same way, or iron if he is sensitive to iron." Needless to say, no very definite beneficial results have hitherto been produced by this beautifully simple process.

